# Cross-Attention Fusion Based Spatial-Temporal Multi-Graph Convolutional Network for Traffic Flow Prediction

**DOI:** 10.3390/s21248468

**Published:** 2021-12-18

**Authors:** Kun Yu, Xizhong Qin, Zhenhong Jia, Yan Du, Mengmeng Lin

**Affiliations:** College of Information Science and Engineering, Xinjiang University, Urumqi 830000, China; ykun@stu.xju.edu.cn (K.Y.); jzhh@xju.edu.cn (Z.J.); 15299182353dy@stu.xju.edu.cn (Y.D.); 18636286649@163.com (M.L.)

**Keywords:** traffic flow prediction, data diversity, cross-attention, spatio-temporal multi-graph

## Abstract

Accurate traffic flow prediction is essential to building a smart transportation city. Existing research mainly uses a given single-graph structure as a model, only considers local and static spatial dependencies, and ignores the impact of dynamic spatio-temporal data diversity. To fully capture the characteristics of spatio-temporal data diversity, this paper proposes a cross-Attention Fusion Based Spatial-Temporal Multi-Graph Convolutional Network (CAFMGCN) model for traffic flow prediction. First, introduce GCN to model the historical traffic data’s three-time attributes (current, daily, and weekly) to extract time features. Second, consider the relationship between distance and traffic flow, constructing adjacency, connectivity, and regional similarity graphs to capture dynamic spatial topology information. To make full use of global information, a cross-attention mechanism is introduced to fuse temporal and spatial features separately to reduce prediction errors. Finally, the CAFMGCN model is evaluated, and the experimental results show that the prediction of this model is more accurate and effective than the baseline of other models.

## 1. Introduction

More and more attention has been paid to traffic flow prediction in intelligent transportation cities. With the rapid economic growth and the increasing number of urban vehicles in recent years, many cities are increasingly troubled by traffic congestion and traffic accidents, which has brought many inconveniences to travel. People all hope to build an intelligent transportation city to alleviate traffic congestion and improve traffic management efficiency. The intelligent transportation system (ITS) has been widely adopted to improve traffic conditions [1,2].

Related research on traffic flow prediction has a history of nearly 40 years, and dozens of forecasting methods have been proposed [3]. According to the prediction time, urban road traffic flow is divided into long-term, medium-long-term, and short-term predictions. The research methods can be divided into classical time series prediction methods, traditional machine learning methods, and deep learning methods from classical time series models, such as historical average (HA) [4] and autoregressive integrated moving average (ARIMA) [5], to traditional machine learning models, such as support vector machine regression (SVR) [6]. Although they can capture the temporal correlation well, they ignore the importance of spatial correlation. It was only with the rise of deep learning models that this problem was solved. In the early stage, researchers mainly used RNN (recurrent neural network), such as LSTM (long short-term memory) [7,8], and GRU (gated recurrent unit) [9,10] models to solve the problem of spatial correlation. Although RNN-based methods can learn spatial correlation, they are often too complex to deal with non-linear correlation. In addition, traditional deep learning methods are easily separated from spatial–temporal correlation and use separate modules to achieve temporal and spatial correlation [11].

**Figure 1:**
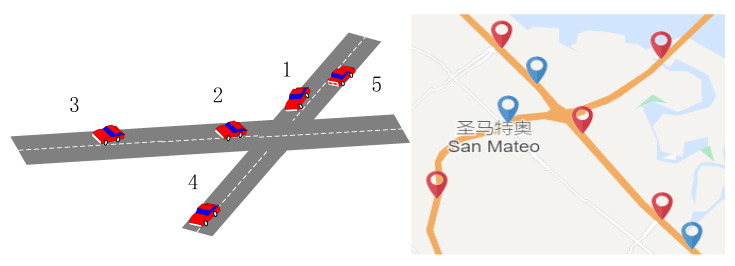
Simulated road intersection.

**Figure 2:**
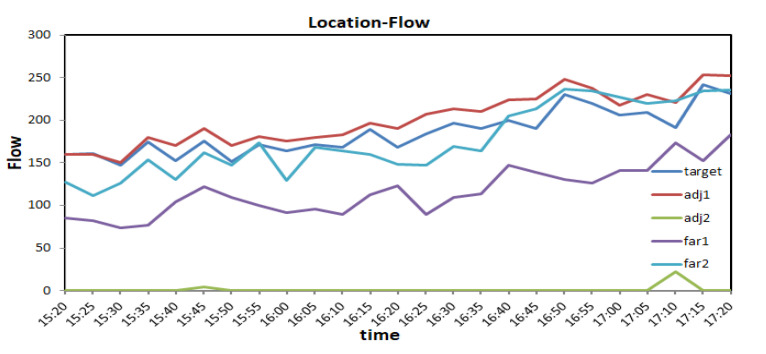
The target is the test node 1, adj1 is adjacent to node 2, adj2 is the reverse adjacent node 5, far1 is the remote node 3, and far2 is the remote node 4.

Recently, the Graph Convolutional Neural Network (GCN) has become the most popular topic in traffic prediction problems [12,13]. Unlike traditional data-driven methods, graph neural networks can process non-Euclidean data and capture road topology information. Compared with other methods, the training speed is faster, and the parameters are also reduced. As shown in Figure 1, a road network is formed at the intersection. When congestion occurs in one section, its adjacent road sections will be significantly affected and spread to other road sections within a certain period. Taking node 1 as the target node, when the traffic jam occurs at node 1, the correlation at neighboring node 2 is strong, while the correlation at adjacent node 5 is weak. Compared with distant node 3 and node 4, they all have a specific correlation. Therefore, it can be seen that the network space correlation between traffic sections is quite complicated. The traffic conditions between two road sections with similar geographical locations may not be correlated, but the traffic conditions between two road sections with a longer distance can be connected. In addition, there is also a specific non-linear correlation between different time observations. Different observations of the same node at different times, such as an hour ago, a day ago, or even one week ago, are correlated to the measured points. To do this, we must incorporate this information into the model to make accurate traffic predictions. Figure 2 is an example of simulated road flow correlation.

We propose a new spatio-temporal fusion model to solve the above problems, called the Cross-Attention Fusion Multi-Graph Convolutional Network (CAFMGCN). This model uses an MCGN and spatio-temporal cross-attention mechanism to study multivariate time series data based on the perspective of a graph. Multi-graph convolution has two functions: one is to construct correlation graphs with three different time attributes to capture temporal features; the other is to construct the spatial semantic correlation graph between the three different roads to capture the spatial features. The input layer takes the historical traffic flow in three different periods, current, daily, and weekly, as the input. We use three temporal graphs to represent the node characteristics of different periods to capture the multi-level temporal correlation. For the convolutional layer, we propose a multi-graph convolutional network to capture the spatial correlation between different nodes and construct three adjacency graphs to express different types of node relationship features to capture spatial correlation and global information. To simultaneously capture the spatio-temporal correlation features in the output layer, we use the cross-attention mechanism to carry out a multi-graph fusion of the constructed spatio-temporal graphs to reduce data loss. The main contributions of this article are as follows:

1. Propose a new multi-graph network model architecture, which separately deals with multi-level temporal correlation (i.e., current, daily, and weekly) and multi-spatial location correlations (i.e., proximity, connectivity, and regional similarity). Modelling is used to capture the temporal and spatial characteristics of nodes at different locations at different times.
2. A new spatio-temporal fusion method is proposed the spatio-temporal cross-attention fusion mechanism. This mechanism can simultaneously capture spatio-temporal features and perform overall fusion, effectively reducing the amount of calculation and data loss when capturing feature graphs.
3. Extensive experiments were conducted on two real traffic data sets. The results show that, compared with the current existing baseline, the CAFMGCN model has better predictability.

## 2. Related Work

This section reviews the latest research on graph convolutional networks and spatio-temporal cross-attention related to traffic flow prediction and points out the limitations of previous research.

### 2.1. Traffic Flow Prediction

In recent years, many excellent achievements have been made in the research on traffic flow prediction. The models used for traffic flow forecasting have evolved from the original traditional time statistical model to today’s deep learning model. As deep learning has made many breakthroughs in speech recognition [14], image classification [15], and other fields, more and more researchers are applying deep learning to spatio-temporal data prediction. For example, literature [16] uses the Recursive Neural Network (RNN) and Convolutional Neural Network (CNN) to model traffic speed to capture the temporal and spatial correlation. Literature [17] proposed a method combining CNN and LSTM, to simulate the changing state of traffic flow, using the interaction between roads to capture spatial correlation. Literature [18] introduced 3D convolution, to automatically capture the correlation of traffic data in spatial–temporal dimensions. Although these existing methods can extract spatial features from the neighborhood of the traffic network, they usually ignore the physical characteristics of the road (for instance, length and speed limit). They are not enough to capture comprehensive road network information. In addition, most of the RNN/CNN models are based on the Euclidean structure to make predictions. They seldom mine the network in non-Euclidean topology, so it cannot characterize the spatial correlation of roads in nature.

### 2.2. Multi-Graph Convolutional Networks

A graph convolutional network is an emerging deep learning model that can deal with non-Euclidean spatial data well and has been applied to spatial modeling of the road network. Literature [19] proposed the Diffusion Convolutional Recursive Neural Network (DCRNN), which modeled traffic flow as a diffusion process on the directed graph and introduced the bidirectional directed graph to consider spatial correlation. Literature [20] uses the combination of graph convolution and gated convolution to capture the spatio–temporal correlation. Because traffic data is constantly changing, in previous GCN methods, the definition of the graph structure is usually partial and static, without considering the dynamic characteristics of the traffic data. For this reason, literature [21] designed an adaptive matrix to consider the changes in influence between nodes and their neighbors. Literature [22] uses a dynamic Laplacian matrix estimator to track the spatial changes between the traffic data. Literature [23] designed the framework of the Attention Graph Convolution Sequence-to-Sequence (AGC-Seq2Seq) model to capture the spatio–temporal changes of traffic patterns in a multi-step prediction method. However, spatio-temporal network data usually shows heterogeneity in both the spatial and temporal dimensions. For example, in an urban road network, observation results recorded by traffic monitoring stations in residential and commercial areas often show different patterns at different times [24]. It is impossible to extract spatial and temporal topological information based on a single GCN.

Multi-graph network models are used in shared bicycle prediction [25] and ride-hailing demand prediction [26], but rarely in road traffic flow prediction. Literature [27,28] models the time diversity through the relationship between the period to be tested and the current, daily, and weekly periods. To capture the long-distance spatio-temporal heterogeneity, literature [29] designed multiple module modeling in different periods. Literature [30] introduced multi-graph GCN to handle three inflow and outflow patterns (current, daily, and weekly) separately, and used high-level spatio-temporal features between different inflow and outflow patterns and between stations nearby and far away, which can be extracted by 3D CNN. Literature [31] uses a multi-graph network to construct an adjacency matrix for different attributes of node adjacency, connectivity, and functionality to measure the spatial correlation between roads. These models can extract temporal and spatial features very well. However, they often separate the spatio-temporal correlation and cannot capture the multi-level temporal and heterogeneous spatial correlations simultaneously.

### 2.3. Cross-Attention Mechanism

The attention mechanism is implemented based on the encoder/decoder model. This model was initially used for machine translation [32], and later literature [33,34] introduced soft attention and hard attention mechanisms in traffic flow prediction. The attention mechanism is used to capture the spatio-temporal correlations of the dynamic changes in the road network, and the global temporal information and spatial correlation are well captured. The literature [35] introduced self-attention into the generative adversarial network and achieved excellent experimental results. The literature [36] introduced the cross-attention module to image detection for the first time, considering the influence of long-distance on the contextual information. It used a more effective method to capture the remote temporal contextual information. The literature [37] proposed an enhanced graph convolutional network based on cross-attention fusion for deep clustering. The literature [38] used cross-attention for ambulance demand prediction. The cross-attention mechanism is not only fast in training, but also takes up very little GPU.

This paper proposes a multi-graph convolution and cross-attention fusion mechanism for traffic flow prediction, to better solve the multi-layer temporal and heterogeneous spatial correlation in the road network.

## 3. Preliminaries

In this section, we define the basic concepts of road traffic network modeling and explain the problems.

**Definition** **1.** *Traffic Road Graph.*

Temporally, we divide the historical period into a set of consecutive time slices, denoted as $T=\left\{h_{t}|t\in 1,2,\cdot \cdot \cdot ,T\right\}$. Each node generates a feature vector on each time slice. This article uses the feature graphs on three historical time slices (e.g., current, daily, and weekly) as input information, elaborated in detail in Section 4.1. 

Spatially, we represent the road graph as a weighted graph G=(V,E,A), where $V=\left\{v_{i}|i\in 1,2,\cdot \cdot \cdot ,N\right\}$ is a set of N detector nodes, and each node $v_{i}$ represents a detector. E is a set of edges connecting these nodes, and each edge $e_{ij}$ represents the correlation between $v_{i}$ and $v_{j}$. The weight of the edge $e_{ij}$ represents the correlation strength between $v_{i}$ and $v_{j}$. The larger the weight, the higher the correlation between the two roads. $A\in R^{N\times N}$ is the adjacency matrix of graph G. This article constructs road graphs from three aspects: road network topology ($X_{w}$), traffic connectivity ($X_{P}$), and regional similarity ($X_{s}$), which will be elaborated in Section 4.2.1.

**Definition** **2.** *Problem Definition.*

We use $x_{t}^{c,i}$ to denote the *c*-th feature of node *i* at time *t*, $X_{t}^{i}$ denotes all eigenvalues of node *i* at time *t*, and $X_{t}$ denotes all eigenvalues of all nodes at time *t*. $X=\left(X_{1},X_{2},\cdot \cdot \cdot ,X_{\tau }\right)$ denotes all the eigenvalues of all nodes on the τ time slices. Given the various historical observations $X_{input}=\left\{X_{t-\tau }|\tau \in \left(0,1,\cdot \cdot \cdot ,w-1\right)\right\}$ of all nodes on the transportation network in the past w time slices, on the premise of $X_{w}$, $X_{P}$, and $X_{s}$, we learn a function *f* by using the model knowledge of the multi-graph network. The traffic flow prediction problem aims to predict the traffic volume of ${\hat{X}}_{t}$ at the next moment. That is:

(1) $${\hat{X}}_{t}=f(X_{w}X_{P}X_{s};\;\left(X_{t-w-1},\cdots ,X_{t-1}X_{t}\right))$$

## 4. Methodology

**Figure 3:**
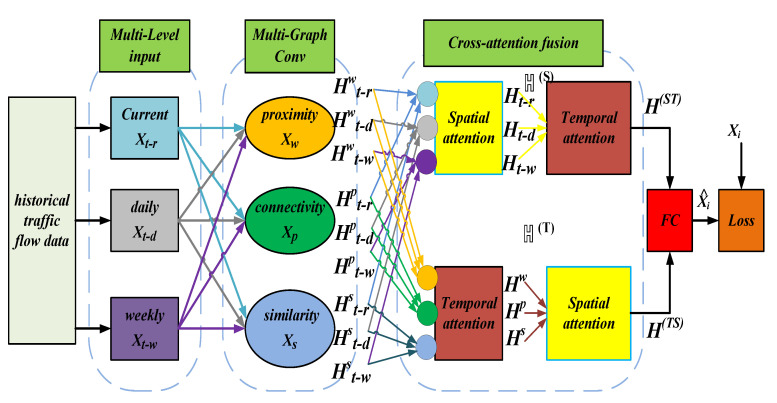
CAFMGCN framework.

Our CAFMGCN model is shown in Figure 3. The model consists of a multi-level temporal input, multi-graph convolution layer, and spatio-temporal cross-attention fusion module.

### 4.1. Multi-Level Temporal Inputs

According to the literature [18,27,28,30], there is a strong correlation between the period to be tested, and its current, daily, and weekly periods. To fully capture the features of the temporal dimension, this paper uses the current, daily, and weekly periods to be tested combined in the temporal dimension according to the temporal sequence as the input of the model, in this way to denote the multi-level temporal correlation.

First, the day is divided into *q* periods on average, and we take the current moment *t* as the starting point; the prediction window size is *p*. Respectively use $X_{r}$, $X_{d}$, and $X_{w}\;$, to denote the temporal dimension feature graphs of the current, daily, and weekly patterns of the period to be tested, then:

(2) $$X_{r}=\left(X_{t-T_{r}+1},X_{t-T_{r}+2},\cdots ,X_{t}\right)\in R^{N\times T_{r}}\;X_{d}=(X_{t-\left(T_{d}/T_{p}\right)*q+1},\cdots ,X_{t-\left(T_{d}/T_{p}\right)*q+T_{p}},X_{t-\left(T_{d}/T_{p}-1\right)*q+1},$$

(3) $$X_{t-\left(T_{d}/T_{p}-1\right)*q+T_{p}},\cdots ,X_{t-q+1},\cdots ,X_{t-q+T_{p}})\in R^{N\times T_{d}}\;X_{w}=(X_{t-7*\left(T_{w}/T_{p}\right)*q+1},\cdots ,X_{t-7*\left(T_{w}/T_{p}\right)*q+T_{p}},X_{t-7*\left(T_{w}/T_{p}-1\right)*q+1},$$

(4) $$X_{t-7*\left(T_{w}/T_{p}-1\right)*q+T_{p}},\cdots ,X_{t-7*q+1},\cdots ,X_{t-7*q+T_{p}})\in R^{N\times T_{w}}$$

**Figure 4:**
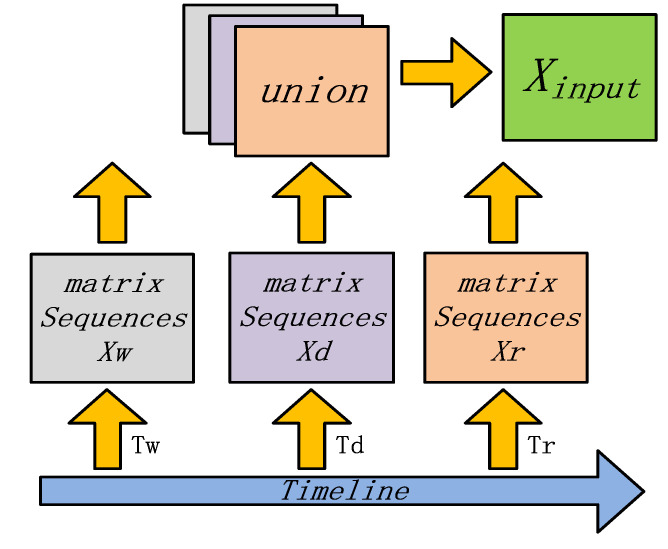
Multi-level temporal model input.

$T_{r}$, $T_{d}$, and $T_{w}$ represent the length of the most current period, the daily period, and the weekly period. The union formed by a mosaic of three tenses is used as the input set of the model:

(5) $$X_{input}=\left[X_{r}\cup X_{d}\cup X_{w}\right]=\{\;X_{t-\tau }|\tau \in \left(1,2,\cdots ,l_{r}\right)\;\cup \;\tau \in \left(d,2d,\cdots ,l_{d}*d\right)\;\cup \;\;\tau \in \left(w,2w,\cdots ,l_{w}*w\right)\;\}$$

where *d* and *w* represent the number of time slices in the daily and weekly time periods (for example, in a 1-h time period, *d* = 24 and *w* = 24 × 7), and $l_{r}$, $l_{d}$, and $l_{w}$ are 3, 1, and 1. The model input is shown in Figure 4.

### 4.2. Multi-Graph Convolutional Layer

To obtain diversified spatial correlation and context information, this paper uses a multi-graph network to capture the heterogeneous spatial correlation. Multi-graph networks can aggregate data in different fields, capture multiple spatial correlations, and learn separately. For example, literature [25,26] modeled spatial correlation from proximity, functional similarity, and connectivity, respectively. The literature [31] uses historical traffic pattern correlation to model heterogeneous spatial. However, they all ignore the impact of the correlation between long-distance and flow on spatial modeling. In this section, we use multiple graphs to encode different correlations between roads and these relationships.

#### 4.2.1. Multi-Graph Construction

Three kinds of correlations between roads were modeled using multiple graphs, including the (1) adjacency graph, encoding space proximity; (2) traffic connectivity graph, considering the connectivity between relatively distant areas; and (3) regional similarity graph, which encodes nodes with similar dynamic directions.

**Figure 5:**
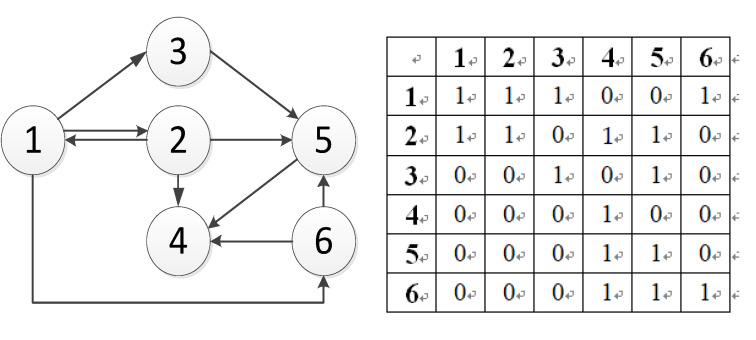
An example of the adjacency matrix.

(1) Traffic Adjacency Graph
  This article defines the traffic adjacency graph ($X_{w}$) based on the spatial proximity, whether there is a straight line between each pair of nodes ($v_{i}\;$,$v_{j}$), sand if $v_{i}$ and $v_{j}$ are connected, then $X_{w,ij}=1$. Otherwise, $X_{w,ij}=0$. The adjacency graph is calculated as follows:
  (6) $$X_{w,ij}=\{\;\;\begin{array}{l} \;1,\;\;v_{i}\;\mathrm{and}\;v_{j}\;\mathrm{are}\;\mathrm{adjacnet}\\ \;0,\;\;\;\;\;\;\;\mathrm{otherwise} \end{array}$$
  Figure 5 shows an example of the adjacency matrix:
(2) Traffic Connectivity Graph
  Since the traffic status is time-series data, the current traffic status on the road will inevitably affect those geographically distant but easily accessible locations. For example, when $X_{ij}$ = 1, $X_{jk}$ = 1, and $X_{ik}$ = 0, nodes *i* and *k* are not directly connected, and information can be transmitted through node *j*. In case of congestion or other accidents, the traffic transmission between non-adjacent node pairs needs to bypass intermediate node pairs to send the congestion information. To ensure whether the data can be transmitted, we judge whether the distant nodes are reachable according to the actual distance. If the node is reachable, the information can be sent; there is a long-distance correlation. Therefore, the traffic connectivity graph defined in this paper is:
  (7) $$X_{p,ij}=\{\begin{array}{l} \;1,\;\;{\bar{v}}_{ij}m-dist_{i,j}\geq 0\\ \;0,\;\;\;\;\;\mathrm{otherwise} \end{array},\;\;\forall_{vi,\;vj}\in V$$
  where ${\bar{v}}_{ij}$ is the average speed between node *i* and *j*, which refers to the average rate of the driver driving without any adverse conditions, and m is the number of time steps moving at the average speed. Thus, *m* determines the element size of $X_{p}$. If the vehicle can travel from node *i* to *j* within *m* time steps, then the element $X_{p,ij}$ = 1, otherwise $X_{p,ij}$ = 0. Intuitively speaking, $X_{p,ij}$ is used to detect whether the vehicle can travel from node *i* to node j at an average speed within a specific number of time steps. Here, set all the diagonal values of $X_{p}$ to 0.
(3) Regional Similarity Graph
  To consider the similarity of different nodes simultaneously, we use the Pearson correlation method to describe them. In previous literature [39,40], the Pearson correlation method mainly analyzes whether the time series are correlated. In contrast, this paper uses the Pearson correlation method to examine whether regional spatial positions are related. In many scenarios, roads with similar spatial locations are not necessarily close in space. For example, both business districts and school districts have identical traffic patterns. Still, when there is a large amount of traffic flow in the business district during the peak hours of workdays, the school district can also have a large amount of traffic flow shortly. It can be seen that different spatial regions have similar positions. Therefore, we use the Pearson correlation method to compose the flow relationship between nodes, which is regarded as the weight $w_{s}\left(i\;,\;j\right)$, and the calculation of $w_{s}\left(i\;,j\right)$ is shown in Equation (8):
  (8) $$w_{s}\left(i\;,j\right)=\frac{\sum_{\tau =1}^{L}\left(x_{i}^{\tau }-\bar{x}\right)\left(y_{j}^{\tau }-\bar{y}\right)}{\sqrt{\sum_{\tau =1}^{L}{\left(x_{i}^{\tau }-\bar{x}\right)}^{2}\sum_{\tau =1}^{L}{\left(y_{j}^{\tau }-\bar{y}\right)}^{2}}}$$
  where $x_{i}^{\tau }$ and $y_{j}^{\tau }$ are the traffic of node *i* and *j* at time *τ*, respectively. * L* is the length of the time series. $\bar{x}$ and $\bar{y}$ are the average traffic of node *i* and *j* in the time length * L*, $w_{s}\left(i\;,j\right)\in$ [0,1]. Then, the regional similarity graph $X_{s}$ can be expressed as Formula (9), where σ=0.5.
  (9) $$X_{s,ij}=\{\begin{array}{l} w_{s}\left(i\;,j\right),\;\;\mathrm{if}\;w_{s}\left(i,\;j\right)\geq \sigma \\ \;0,\;\;\;\;\mathrm{otherwise} \end{array}$$
  We denote the above three feature maps as a set θ:
  (10) $$\theta \in \left(X_{w},X_{P},X_{s}\right)$$

#### 4.2.2. Multi-Graph Convolutional Network

To capture the diversity and heterogeneity spatial correlation, we adopted the Multi-Graph Convolutional Network (MGCN) model, which consists of several separate graph structures and inputs the characteristics of each node with different spatial position relationships into one separate graph, and then use graph convolution based on spectrum theory [22] to analyze graph topology on time slices. In graph analysis, the superposition of the GCN layer and 1-order filter can achieve an effect similar to that of the k-order Chebyshev polynomial filter [41], which improves the training speed and enhances the prediction accuracy. The layering propagation law of Chebyshev polynomials [28] is:

(11) $$H=\mathrm{ReLU}\left(\sum_{k=0}^{K}\tilde{L}XW_{k}\right)$$

where $H\in R^{u\times 1}$,$\;X\in R^{v\times 1}$, and $W_{k}\in R^{v\times u}$ denote the hidden layer, input feature vector, and the trainable parameter matrix extracted in operation; ReLU is the activation function; and $\tilde{L}\in R^{v\times v}$ is the rescaled Laplace matrix, $\tilde{L}=\frac{2}{\lambda_{\max}}L-I_{N}$, where $L=I_{N}-D^{-\frac{1}{2}}AD^{-\frac{1}{2}}$ is the symmetric normalized Laplacian graph, and $\lambda_{\max}$ is its maximum eigenvalue. $I_{N}$ is the identity matrix, A is the adjacency matrix, and *D* is the degree matrix. The propagation law can be considered as a spectral filter in the Fourier domain. Each road section inputs three GCNs and three feature matrices generated by the corresponding road graph. The propagation law of the 1-order GCN layer defined in this paper is:

(12) $$H^{l+1}=\mathrm{ReLU}(\tilde{D}-\frac{1}{2}\tilde{X}\tilde{D}-\frac{1}{2}H^{l}W^{l})$$

where $\tilde{X}\in R^{v\times v}$ is the adjacency matrix determined by the topological graph, $\tilde{D}$ is the diagonal degree matrix of $\tilde{X}$, $H^{l}$ is the characteristic matrix of layer L, and $W^{l}$ is the parameter matrix of layer L.

### 4.3. Cross-Attention Mechanism Fusion

**Figure 6:**
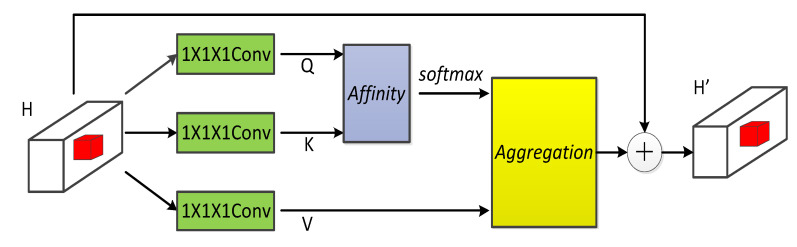
Cross-attention model. Q, K, and V are all extracted feature graphs.

Although multiple graphs can be used as input, how to effectively integrate temporal and spatial information simultaneously is a new problem in the current stage of research. In literature [42], spatio-temporal features are fused by the matrix multiplication of a spatio-temporal fusion graph. The literature [43] directly merges all the features by summing and integrating the generated topological graphs. These methods cannot support the fusion of multiple temporal and multiple spatial information simultaneously. To effectively fuse the correlations between the adjacency graph, connected graph, and regional similarity graph on multi-layer temporal slices, we propose a dynamic fusion method called the cross-attention fusion mechanism. The principle of cross-attention fusion is to use the most basic attention mechanism to capture information, simultaneously, from the temporal and spatial perspective in an interlaced manner. Figure 6 shows a general model of the cross-attention mechanism.

#### 4.3.1. Cross Attention

We take the multi-layer temporal input ($X_{t-\tau }\in X_{input}$ (Equation (5)) in parallel through the multi-graph feature set θ (Equation (5)), to get the hidden spatio-temporal representations ℍ.

(13) $$ℍ=\left\{H_{t-\tau }^{\theta }\in R^{u}|\theta \in \left(X_{W},X_{P},X_{s}\right)\tau \in T_{h}\right\}$$

**Figure 7:**
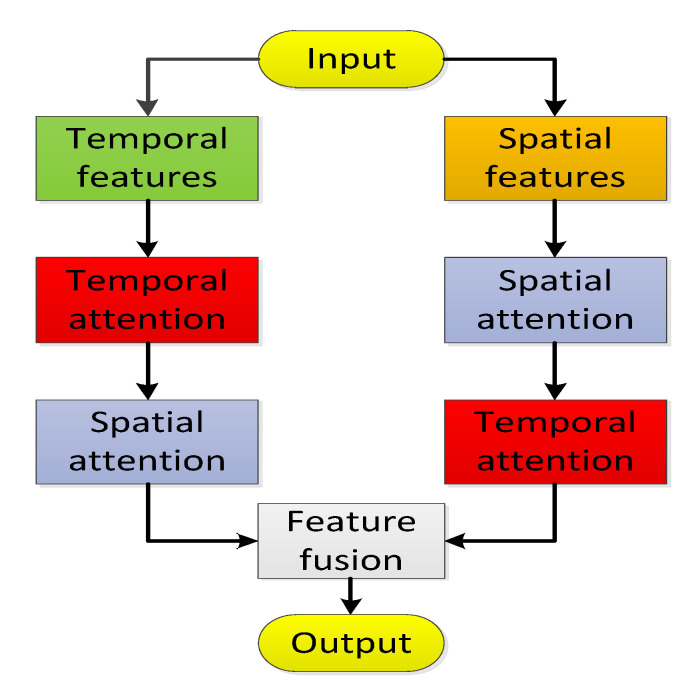
Flow Chart of the Cross Attention.

Here, $H_{t-\tau }^{\theta }\in ℍ$, its superscript *θ* carries spatial correlation information and the subscript *t-τ* carries temporal correlation information. To feature the fusion of spatio-temporal information, we use a spatio-temporal attention mechanism in two steps, as shown in Figure 7.

#### 4.3.2. Feature Fusion

In the first step, we divide the spatio-temporal representation $H_{t-\tau }^{\theta }$ into two expression forms: (1) according to the same temporal but different spatial locations into $H_{t}^{X_{w}}$, $H_{t}^{X_{p}}$, and $H_{t}^{X_{s}}$; (2) according to the same spatial but different temporal information into $H_{t-r}^{\theta },\;H_{t-d}^{\theta },\;{\mathrm{and}H}_{t-w\;}^{\theta }$. The former represents the spatial features of heterogeneous multi-graphs and is called spatial attention; the latter represents multi-level temporal features and is called temporal attention. Therefore, the spatial attention (Equation (14)) and temporal attention (Equation (15)) of the first step are as follows:

(14) $$\{\begin{array}{l} \alpha^{1s}=softmax\left(H_{t-\tau }^{\theta }W_{H}^{1s}+G^{\theta }W_{G}^{1s}+b^{1s}\right)\\ H_{t-\tau }^{\left(S\right)}=\overset{N}{\underset{\theta }{\sum }}\alpha_{\theta }^{1s}\cdot H_{t-\tau }^{\theta },\;\;\;\;\;\;\;\;\;H_{t-\tau }^{\left(S\right)}\in {ℍ}^{\left(S\right)} \end{array}$$

(15) $$\{\begin{array}{l} \alpha^{1t}\;=softmax\left(H_{t-\tau }^{\theta }W_{H}^{1t}+M_{t-\tau }W_{M}^{1t}+b^{1t}\right)\\ H_{\theta }^{\left(T\right)}=\overset{T_{h}}{\underset{\tau }{\sum }}\alpha_{\tau }^{1t}\cdot H_{t-\tau }^{\theta },\;\;\;\;\;\;\;\;\;H_{\theta }^{\left(T\right)}\in {ℍ}^{\left(T\right)} \end{array}$$

where $W_{H}^{1t}$,$W_{M}^{1t}$,$W_{H}^{1s}\;$, and $W_{G}^{1s}$ denotes trainable parameters; $b^{1t}$ and $b^{1s}$ denotes deviation vectors; and $\alpha_{\tau }^{1t}$ and $\alpha_{\theta }^{1s}$ denotes normalized weight scalars, namely $\overset{T_{h}}{\underset{\tau }{\sum }}\alpha_{\tau }^{1t}=\overset{N}{\underset{\theta }{\sum }}\alpha_{\theta }^{1s}=1$, where $\alpha_{\tau }^{1t}\in \left(0,1\right)$ and $\alpha_{\theta }^{1s}\in \left(0,1\right)$. $M_{t-\tau }$ represents the density of the time slice $h_{t-\tau }$, and $G^{\theta }$ denotes a succinct vector of the graph θ.

**Figure 8:**
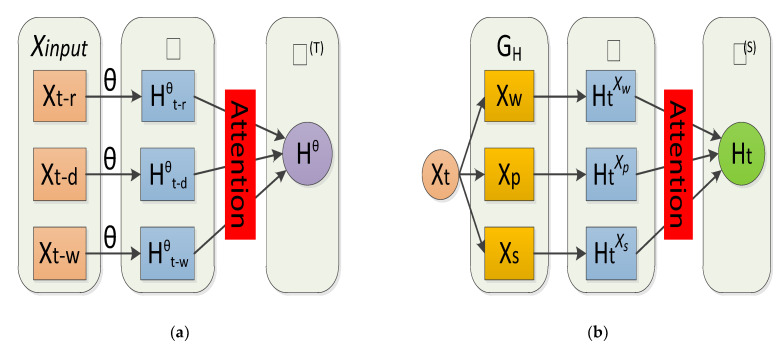
First step cross attention. (**a**) Time series: Temporal Fusion. (**b**) Multi-Graph: Spatial Fusion.

The second step, as shown in Figure 8, due to the first step being in a set of temporal attention, produces a spatial set that incorporates temporal information ${ℍ}^{(T)}=\left\{H_{\theta }^{\left(T\right)}|\theta \in \left(X_{w},X_{P},X_{s}\right)\right\}$. Spatial attention generates a temporal set that contains spatial information ${ℍ}^{(S)}=\left\{H_{t-\tau }^{\left(S\right)}|\tau \in T_{h}\right\}$; we then use cross-attention to perform temporal attention on the newly fused spatial set ${ℍ}^{(S)}$ and perform spatial attention on the newly fused temporal set ${ℍ}^{(T)}$ to get a new set of equations.

(16) $$\{\begin{array}{l} \alpha^{2t}\;=softmax\left(H_{t-\tau }^{\left(S\right)}W_{H}^{2t}+M_{t-\tau }W_{M}^{2t}+b^{2t}\right)\\ H^{\left(\mathrm{ST}\right)}=\overset{T_{h}}{\underset{\tau }{\sum }}\alpha_{\tau }^{2t}\cdot H_{t-\tau }^{\left(S\right)} \end{array}$$

(17) $$\{\begin{array}{l} \alpha^{2s}\;=softmax\left(H_{\theta }^{\left(T\right)}W_{H}^{2s}+G^{\theta }W_{G}^{2s}+b^{2s}\right)\\ H^{\left(\mathrm{TS}\right)}=\overset{N}{\underset{\theta }{\sum }}\alpha_{\theta }^{2s}\cdot H_{\theta }^{\left(T\right)} \end{array}$$

The notation here is similar to the formula notation in the first step. The principle of cross-attention mechanism fusion is to simultaneously represent multi-layer temporal correlation and heterogeneous spatial correlation as two views, and then perform cross-fusion. Equations (14) and (16) compress ${ℍ}^{\left(S\right)}$ into $H^{\left(\mathrm{ST}\right)}$ based on spatial continuity, and Equations (15) and (17) compress ${ℍ}^{\left(T\right)}$ into $H^{\left(\mathrm{TS}\right)}$ based on temporal continuity. Finally, input the two compressed matrices into a fully connected layer to get the final prediction result, which is:

(18) $${\hat{X}}_{t}=tanh\left(H^{\left(\mathrm{TS}\right)}W_{TS}+H^{\left(\mathrm{ST}\right)}W_{ST}+b\right)\;$$

Among them, $W_{TS}$ and $W_{ST}$ are trainable parameters, and *b* is biased.

## 5. Experiment

### 5.1. Datasets Description

We downloaded two California traffic data sets, PeMS04 and PeMS08, on the official website (https://pems.dot.ca.gov/) (accessed on 23 May 2021) and GitHub. Traffic data is collected in real-time every 30 s and aggregated every 5 min [41]. Three traffic measurements were considered in our experiment: total flow, average speed, and distance. We use 1 h ($T_{p}$ = 12) as the historical time window to predict the traffic conditions in the future, 15/30/45/60 min.

PeMSD4 contains 3848 detectors on 29 roads. We selected 307 sensors and collected data for two months, from 1 January to 28 February 2018.

PeMSD8 contains 1979 detectors on 8 roads. We selected 170 sensors and collected data for two months, from 1 July to 31 August 2016. Table 1 summarizes some critical information data of these two data sets.

### 5.2. Settings

Use Pytorch to implement our model. First, set the input time parameter to $T_{r}=T_{p}\times 3$, $T_{d}=T_{p}\times 1$, and $T_{w}=T_{p}\times 1$, where $T_{p}=12$ is the prediction window size. We captured three types of positional relationships, so *N* = 3. In the multi-graph convolution stage, the graph and temporal convolution kernel size are set to 64 and 3. In the training process, we selected the best batch_size = 32, learn_rate = 1 × 10^−3^, and epoch = 100. All experiments were compiled and tested on a Windows System (CPU: Intel(R) Core(TM) i5-5200U CPU @2.20 Ghz) using Xshell and WinSCP to connect to the server (GTX 1080 Ti).

### 5.3. Baselines

We compare CAFMGCN with the following eight baselines:

- SVR: Support Vector Regression uses a linear support vector machine for regression tasks [6].
- GRU: Gated Recurrent Unit network, a special kind of RNN [10].
- DCRNN: Diffusion Convolution Recurrent Neural Network is a data-driven prediction framework with a diffusion recurrent neural network to capture spatio-temporal dependence [19].
- STGCN: Spatio-Temporal Graph Convolutional Networks is an integrative framework of graph convolution network and convolutional sequence modeling layer for modeling spatial and temporal dependencies [20].
- Graph WaveNet: a framework that combines the adaptive adjacency matrix into graph convolution with 1D dilated convolution [21].
- ASTGCN: Attention Based Spatial-Temporal Graph Convolutional Networks introduce spatial and temporal attention mechanisms into a model. Only the most recent components of the modeling period are used to maintain a fair comparison [28].
- STSGCN: Spatial-Temporal Synchronous Graph Convolutional Networks, which utilizes localized spatio-temporal subgraph module to independently model the local correlation [29].
- STFGNN: Spatial-Temporal Fusion Graph Neural Networks could effectively fuse various spatio-temporal graphs in different periods, in parallel. We compare the fusion methods of this model [42].

### 5.4. Evaluation Metric

Three evaluations are used as evaluation: mean absolute error (MAE), mean fundamental percentage error (MAPE), and root mean square error (RMSE).

### 5.5. Experiment Results Analysis

**Figure 9:**
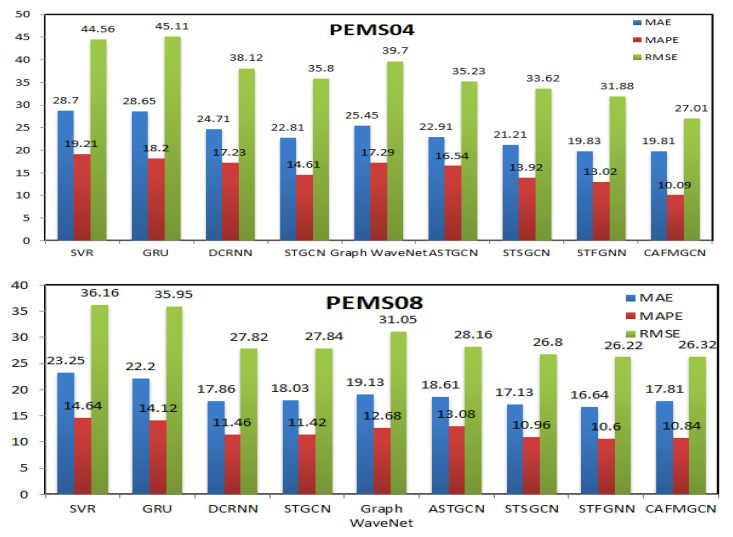
Average prediction results of different methods.

This paper compares eight baseline models with our model. It can be seen, from Figure 9, that our CAFMGCN model has achieved the best results compared with other models on the three evaluation indicators of MAE, MAPE, and RMSE. The traditional time series methods SVR and GRU only consider temporal correlation, ignoring the importance of spatial correlation, so the prediction effect is not ideal. Based on deep learning methods, DCRNN, STGCN, Graph WaveNet, ASTGCN, STSGCN, STFGNN, and our model, CAFMGCN, graph structure and graph topology is introduce to capture spatial information and achieve better prediction results. Graph WaveNet has the worst prediction effect because it only uses 1D CNN and cannot stack its spatio-temporal layers and expand the receptive field. DCRNN, STGCN, and ASTGCN, respectively, use two modules to deal with temporal and spatial correlations, ignoring the heterogeneity of spatio-temporal data, and the prediction effect is average. However, STSGCN and STFGNN simultaneously process temporal and spatial correlation, with higher MAE, MAPE, and RMSE, but ignore temporal diversity. Our CAFMGCN considers the diverse temporal and heterogeneous spatial correlations and, simultaneously, captures spatio-temporal correlations and performs multi-graph fusion. The experimental results show that CAFMGCN can better capture the heterogeneous spatio-temporal correlation of the road network, thus achieving the best prediction effect.

**Figure 10:**
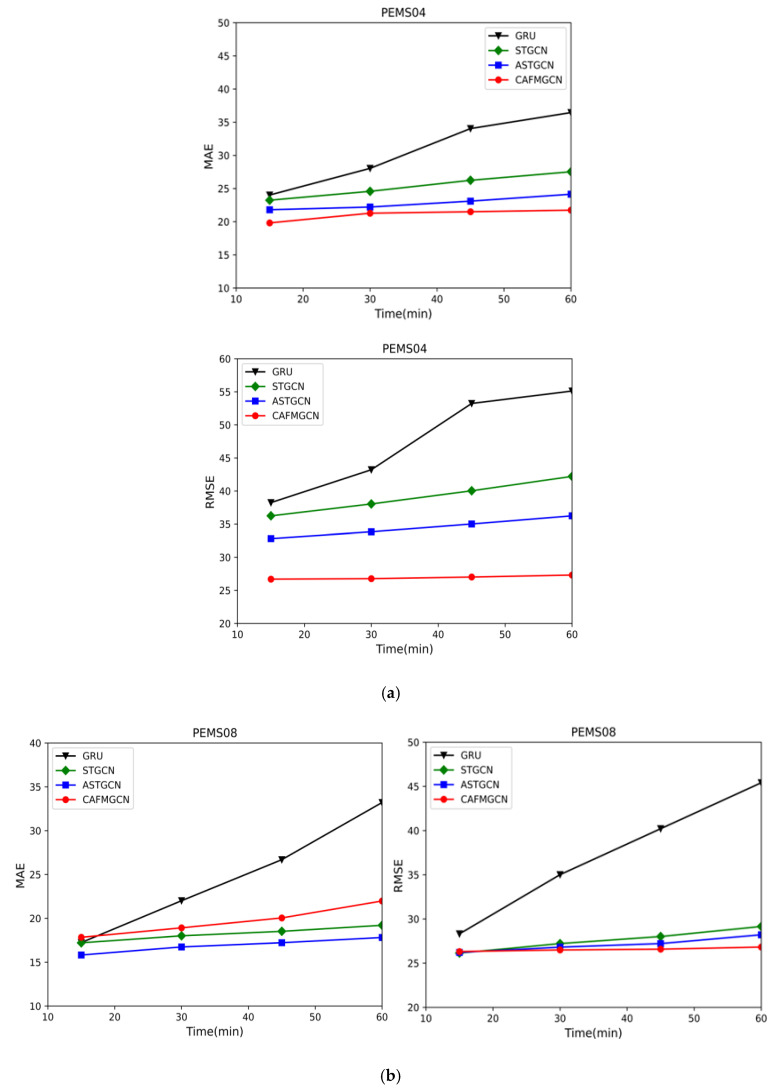
Comparison of prediction performance under different periods. (**a**) PeMSD4. (**b**) PeMSD8.

Figure 10 shows the traffic flow forecasts for the next 15, 30, 45, and 60 min of the two data sets. Taking GRU, STGCN, and ASTGCN as the baseline, it can be seen from the figure that, as time increases, each model’s prediction shows an upward trend, but the prediction error of our model rises more slowly than the other three models, because we consider the long-distance time correlation and combine the features of multi-graphs to reduce the model’s prediction error. Effective forecasting results have been achieved in the short term, and are very helpful for long-term forecasting.

### 5.6. Ablation Experiment

To verify the multi-graph heterogeneity and cross-attention mechanism, we conducted ablation research on PEMS04 as an example. For heterogeneity, we use a single variable method to reduce the heterogeneity of multiple graphs with a single chart based on three single graph experiments, namely, adjacency graph, connectivity graph, and regional similarity graph. For cross-attention, we use the matrix multiplication method mentioned in literature [31], to represent multi-graph fusion and GRU model for experiments.

**Figure 11:**
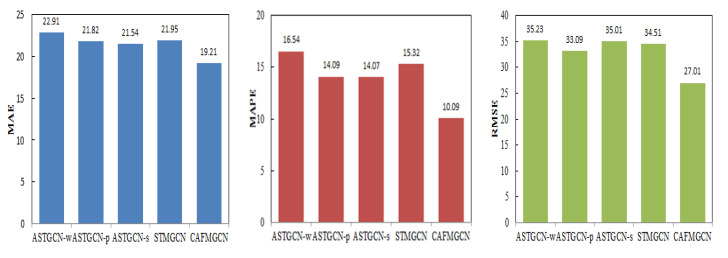
Comparison of average performance of different models of PEMS04.

As shown in Figure 11, the prediction effect of single-graph ASTGCN-w, ASTGCN-p, ASTGCN-s, and the multi-graph non-attention mechanism is not as good as that of CAFMGCN, indicating that the effectiveness of the multi-graph and the fusion effect of the cross-attention mechanism are better.

## 6. Conclusions and Outlook

In this paper, we propose a new model CAFMGCN for traffic flow prediction. The model uses multi-graph GCN to process multi-level temporal correlation, encode the non-Euclidean correlation between heterogeneous spatial roads, and fuse MGCN with cross-attention to capture hidden temporal and spatial information. The combination of a multi-graph convolution module and cross-attention mechanism can capture the dynamic spatio-temporal characteristics of traffic data simultaneously. The experiments based on two real traffic data sets prove that our model CAFMGCN can achieve better performance.

The following two issues are mainly considered in the future: weather factors have always been one of the challenges faced by traffic flow forecasting. The environment dramatically influences travel, which needs to be observed based on specific weather data. Furthermore, significant events, such as festivals, holidays, and concerts, are often encountered in life, which can easily cause traffic jams. Solving these problems will further improve the transportation system.

## Figures and Tables

**Table 1:** Dataset description and statistics.

Datasets	#Nodes	#Edges	#Time Steps	#Missing Ratio
PEMS04	307	340	16992	3.182%
PEMS08	170	295	17856	0.696%

## Data Availability

Data link is https://github.com/yukun-master/CAFMGCN (accessed on 14 December 2021).
